# Plasmablastic Lymphoma Masquerading as Plasma Cell Myeloma: A Case Report

**DOI:** 10.7759/cureus.77310

**Published:** 2025-01-12

**Authors:** Mouad Harandou, Mohammed Bensalah, Rachid Seddik

**Affiliations:** 1 Hematology, Mohammed VI University Hospital, Mohammed First University, Oujda, MAR

**Keywords:** lymphoma, malignant hematology, multiple myeloma, plasmablastic, plasmablastic large b-cell lymphoma

## Abstract

Plasmablastic lymphoma (PBL) is an uncommon and aggressive type of B-cell lymphoma, and it primarily affects individuals with compromised immune systems, particularly human immunodeficiency virus (HIV)-positive patients. It has a strong predilection for the oral cavity and a high association with Epstein-Barr virus (EBV) infection, with only rare extra-oral and EBV-negative cases. Tumor cells in PBL are represented by immature plasma cells known as plasmablasts, a morphological appearance shared with many B-cell and plasma cell neoplasms, which makes the distinction very challenging among these entities, especially in extra-oral locations and EBV-negative cases. PBL is characterized by an aggressive clinical outcome, with a very poor prognosis. Here, we describe an unusual case involving a 72-year-old patient who was previously diagnosed with plasma cell myeloma, which exhibited unusually aggressive and refractory progression; histomorphological investigations were consistent with an extra-oral, EBV-negative PBL. The rapid disease progression and lymph node involvement along with the presence of plasmablastic morphology raised the suspicion of a PBL. Throughout this case, we highlight the importance of a complete histopathological and immunohistochemical examination with correlation with clinical manifestations to ensure a precise diagnosis, particularly in extra-oral, EBV-negative cases.

## Introduction

Plasmablastic lymphoma (PBL) is a rare and aggressive non-Hodgkin lymphoma [1]. It is a B-cell lymphoma that affects primarily the elderly population with a high prevalence for immunocompromised patients, especially those infected with human immunodeficiency virus (HIV). It has a high predilection for the oral cavity [2]. This lymphoma is characterized morphologically by a diffuse proliferation of immature plasma cells known as plasmablasts, admixed with immunoblasts; the neoplastic cells are characterized by the expression of plasma cell markers and typically the non-expression of B-cell-specific markers [3]. It represents a potential differential diagnosis with plasmablastic myeloma, plasma cell myeloma, and other B-cell lymphomas with plasmablastic differentiation such as anaplastic lymphoma kinase (ALK)-positive large B-cell lymphoma, human herpesvirus 8 (HHV-8)-associated large B-cell lymphoma, and primary effusion lymphoma [4]. PBL is characterized by distinct genetic alterations such as myelocytomatosis (MYC) viral oncogene homolog gene overexpression and signal transducer and activator of transcription 3 (STAT3) activation [2]. Distinguishing between potential differentials is crucial since PBL is characterized by a worse prognosis and an aggressive clinical outcome compared to other large B-cell lymphomas [2]. The majority of PBL cases show co-infection with Epstein-Barr virus (EBV), and the identification of EBV, the absence of ALK expression, and HHV-8 negativity represent a significant clue to establish the diagnosis and to rule out EBV-non-associated hematologic neoplasms with similar morphology [3]. Throughout this case report, we highlight the unusual clinical presentation of an extra-oral PBL, without the expression of EBV, falsely diagnosed initially as a plasma cell myeloma.

## Case presentation

Here is the case of a 72-year-old patient who was initially diagnosed with a plasma cell myeloma, based on bone marrow infiltration by mature-appearing plasma cells and the presence of light-chain restriction. The patient's initial symptoms included pallor and chronic fatigue, without reported lymph node or spleen enlargement during the previous physical examination. The patient lacked a history of HIV infection or immunosuppressive therapy. Monoclonal gammopathy was detected through protein electrophoresis, further supporting the diagnosis of plasma cell myeloma. During medical treatment, the patient experienced significant general deterioration and enlargement of multiple lymph nodes, with no clinical signs of remission.

**Figure 1:**
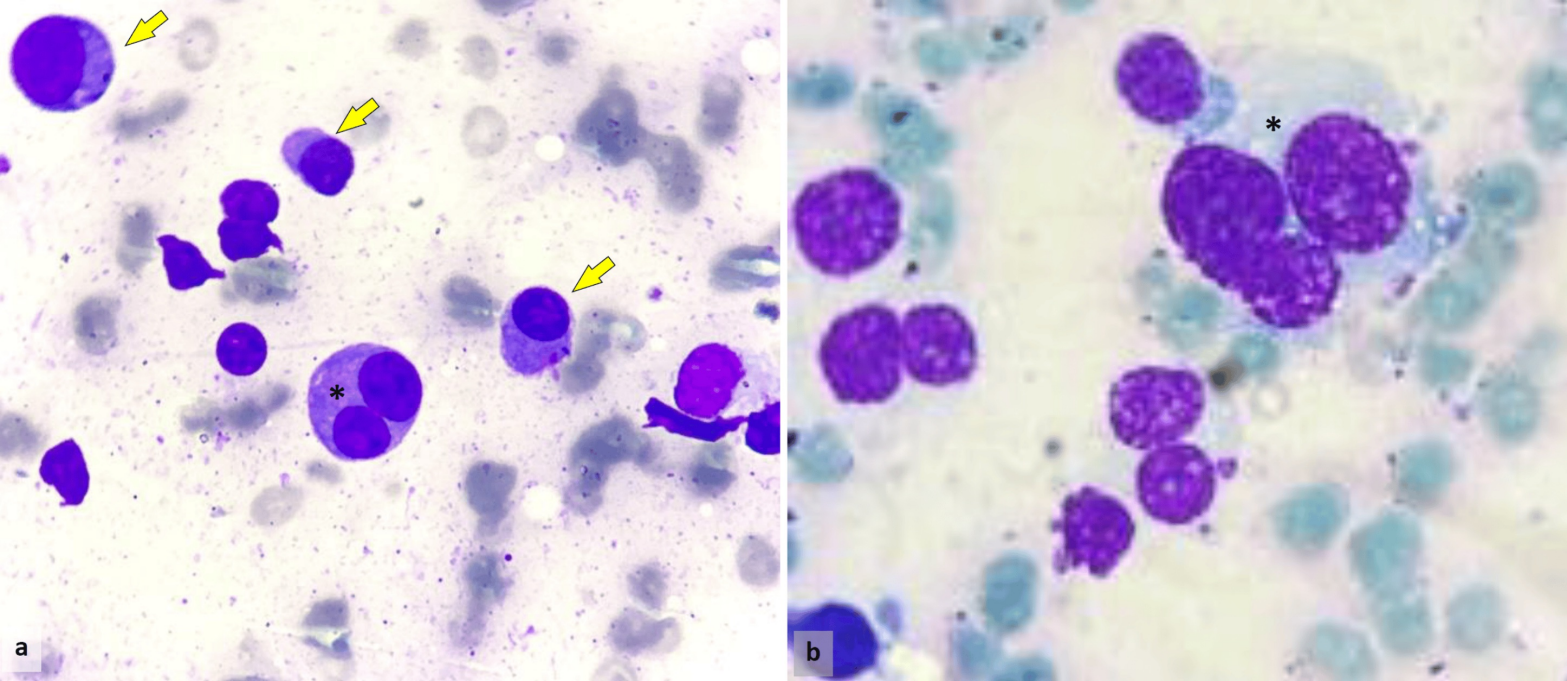
Bone marrow aspiration showing multiple plasmacytoid cells with eccentric, enlarged nuclei (arrow) and multinucleation (*) (a, b: MGG stain, ×400). MGG: May-Grünwald Giemsa

A follow-up bone marrow aspiration revealed markedly dysplastic plasma cells, comprising nearly 70% of total cellularity, exhibiting features suggestive of a plasmablastic differentiation (Figure 1).

The electrophoresis and immunofixation showed a kappa-type light-chain restricted gammopathy, as demonstrated initially. The blood count showed anemia (hemoglobin: 6 g/dl, normal range: 12-16 g/dl), leukocytopenia (white blood cells: 2500/μl, normal range: 4000-11,000/μl), and thrombocytopenia (platelet: 30,000/μl, normal range: 150,000-450,000/μl). The blood smear did not show any abnormal cells. HIV infection was excluded.

**Figure 2:**
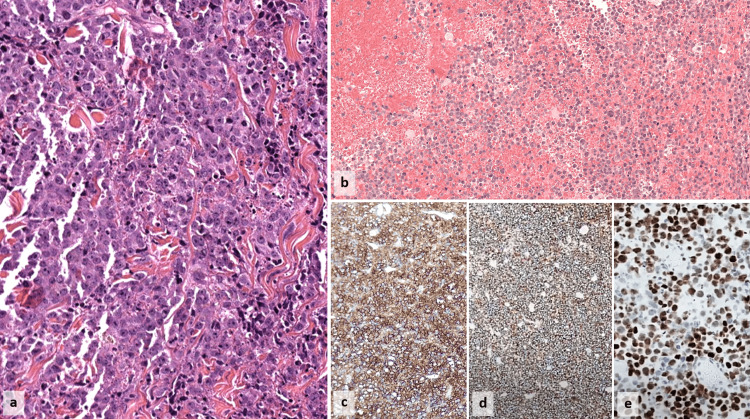
Photomicrographs of the lymph node show diffuse infiltration by plasmablastic cells with enlarged, centrally located nuclei and prominent nucleoli (a: H&E, ×200). The bone marrow biopsy reveals multiple immature large plasma cells with a plasmablastic appearance (b: H&E, ×100). Immunohistochemical staining of the lymph node proliferation shows the diffuse positivity of plasmablastic cells for both CD79a (c: ×100) and CD138 (d: ×40), along with an elevated Ki-67 index (e: ×200). H&E: hematoxylin and eosin

A CT scan was performed revealing multiple lymph nodes at the thoracic and cervical levels. Subsequently, a cervical lymph node resection was performed, revealing on the hematoxylin and eosin (H&E)-stained histology section a lymphomatous proliferation composed of plasmablastic cells arranged in diffuse sheets. These cells showed rounded and eccentrically placed nuclei with evident nucleoli and eosinophilic cytoplasm. Immunohistochemical analysis confirmed the diffuse expression of CD45, CD138, and CD79a, along with kappa light-chain restriction. Notably, tumor cells did not express CD20, CD30, CD117, PAX5, ALK, EBV, or HHV-8. The proliferative index, assessed by Ki-67, was unusually high at 80%, diverging from the typical profile of plasma cell myeloma (Figure 2).

Given the extensive lymph node involvement, the absence of sufficient criteria for a plasma cell myeloma including no osteolytic lesions or hypercalcemia, and the immunohistochemical findings, the diagnosis was revised to PBL. Unfortunately, the patient succumbed to the disease shortly thereafter, prior to receiving the accurate diagnosis and appropriate treatment.

## Discussion

PBL is an uncommon B-cell lymphoma, accounting for approximately 1% of all large B-cell lymphomas [5]. It has been recently described by the World Health Organization classification of hematologic malignancies [6]. This variant is more aggressive with frequent relapse and a poor prognosis [2]. It predominantly affects men, representing almost 75% of patients [3], and is more common in adults [2]. Typically, it manifests in extranodal sites, with the oral cavity, particularly the gingiva and palate, being the most commonly affected. Other locations have also been reported, with a lesser frequency such as the gastrointestinal tract, genitourinary system, skin, and bone [7], while solitary lymph node and marrow involvement is uncommon [2]. Although PBL primarily occurs in HIV-positive patients, cases in HIV-negative individuals have been reported, often associated with immunosuppression due to solid organ transplant [8], and this association is not obligatory since few cases of PBL have been described in immunocompetent patients as well [3]. In our case, the patient lacked a history of HIV infection or immunosuppressive therapy; however, advanced age-related immunosenescence may pose a potential risk factor.

Examination of blood smear and marrow aspiration may reveal the presence of tumor cells exhibiting plasmablastic and immunoblastic morphology, featuring possible multinucleations, evident mitotic activity, and apoptotic tumor cells [2]. Histologically, PBL shows neoplastic cells with eccentric vesicular nuclei with prominent nucleoli and abundant cytoplasm; mitotic figures are often prominent with multiple tingible body macrophages [1]. PBL involving nodal sites typically exhibits a diffuse plasmacytic appearance, with cells displaying a more mature morphology characterized by smaller size and clumped nuclear chromatin. In our case, the neoplastic cells in both peripheral blood smear and marrow aspiration showed a prominent mature plasmacytic appearance. The bone marrow biopsy is useful to evaluate disease extension and marrow involvement [2]. Neoplastic cells usually have a plasma cell immunophenotype, with the diffuse expression of plasma cell markers including CD38, CD138, Blimp1, MUM1, XBP1, and VS38c, while showing absent or faint staining for B-cell-specific markers, such as CD20 and PAX5. Notably, unlike plasma cell neoplasms, PBL often exhibits MYC overexpression [9]. Despite the strong association of PBL with EBV infection, which helps differentiate PBL from myeloma [1], cases with negative EBV have also been documented representing almost 40% of cases [4]. The lack of EBV expression within the neoplastic cells posed a considerable diagnostic challenge.

PBL must be differentiated from other hematologic neoplasms with plasmacytic differentiation due to differences in treatment approaches and prognosis [8]. The differentials include plasma cell myeloma, ALK-positive large B-cell lymphoma, HHV-8-associated large B-cell lymphoma, and primary effusion lymphoma. The distinction can be particularly challenging, especially in EBV-negative and extra-oral cases, as PBL often shares morphological similarities with these entities. Therefore, reaching a definitive diagnosis relies on careful correlation with clinical and imaging findings [7].

Plasma cell myeloma is characterized by the presence of renal disease, paraprotein, osteolytic lesions, hypercalcemia, and bone marrow involvement, rarely manifests in extramedullary and nodal locations [7], and lacks a confirmed association with HIV and EBV infections [8]. Plasmablastic myeloma is suggested when plasmablastic morphology is observed in over 30% of tumor cells. CD56 and CD10 expression can also be observed in plasmablastic myeloma, plasma cell myeloma, and PBL, which diminishes their diagnostic utility [4]. Consistently, the proliferation index (Ki-67) is often high exceeding 80% [2], aligning with our case. The immunoblastic variant of large B-cell lymphoma exhibits morphological similarities to PBL but can be distinguished by its diffuse expression of B-cell markers, including CD19, CD20, and CD22, which are typically absent in the majority of PBL cases. ALK-positive large B-cell lymphoma also lacks CD20 expression, with no association with EBV; however, ALK positivity in tumor cells serves as a diagnostic criterion [10]. On the other hand, HHV-8-associated large B-cell lymphoma and primary effusion lymphoma are characterized by their positivity for HHV-8 [8]. Differentiating between these entities is crucial as PBL often shows a less favorable prognosis. Approximately 50% of PBL cases exhibit MYC alterations; however, its concomitant association with plasma cell myeloma and plasmablastic myeloma reduces its diagnostic utility [11].

Survival rates for PBL remain low, with an overall five-year survival rate lower than 30% [8]. Most patients succumb shortly after the onset of symptoms [6]. Treatment protocols for PBL are not well-established, and various chemotherapy regimens are employed, often in conjunction with antiretroviral therapy for HIV and EBV infections [6]. Occasionally, intensive chemotherapy may be utilized, although its superiority over standard protocols has not been conclusively demonstrated, necessitating further research into the efficacy of different treatment approaches [8].

Given the rapid progression of the disease, its aggressive clinical manifestation involving the bone marrow and lymph nodes, the absence of criteria indicative of a plasma cell myeloma, the markedly elevated proliferative index, and the negative staining for HHV-8 and B-cell markers, the diagnosis of PBL was strongly favored in our case.

## Conclusions

PBL remains an aggressive lymphoma with an unfavorable prognosis. It shares many similarities with other hematologic neoplasms. The plasmablastic morphology is important when considering a PBL; however, clinical presentation and immunohistochemical analysis remain crucial excluding potential alternative diagnoses. EBV positivity within neoplastic cells, concurrent HIV infection, and a high proliferative index support the diagnosis of PBL. Throughout this case, we highlight the importance of a meticulous morphological examination of plasma cells to search for any plasmablastic differentiation that may trigger suspicions of PBL.
